# Evaluating the predictive value of late gadolinium enhancement assessed by cardiac magnetic resonance on sudden cardiac death in patients selected for implantable cardioverter defibrillator and cardiac resynchronization therapy implantation: a systematic review and meta-analysis

**DOI:** 10.1007/s00392-024-02441-2

**Published:** 2024-04-08

**Authors:** Richárd Masszi, Előd-János Zsigmond, Réka Ehrenberger, Caner Turan, Péter Fehérvári, Brigitta Teutsch, Zsolt Molnár, Zsófia Drobni, Hajnalka Vágó, Péter Hegyi, Béla Merkely, Annamária Kosztin

**Affiliations:** 1 Centre for Translational Medicine, Semmelweis University, Budapest, 1085 Hungary; 2 Heart and Vascular Center, Semmelweis University, 68 Városmajor Street, Budapest, 1122 Hungary; 3 Department of Cardiology, Military Hospital – State Health Centre, Budapest, Hungary; 4 Doctoral School of Clinical Medicine, University of Szeged, Szeged, Hungary; 5 Department of Anesthesiology and Intensive Therapy, Semmelweis University, Budapest, Hungary; 6 Department of Biostatistics, University of Veterinary Medicine, Budapest, Hungary; 7 Institute for Translational Medicine, Medical School, University of Pécs, Pécs, 7623 Hungary; 8 Department of Sports Medicine, Semmelweis University, Budapest, Hungary; 9 Institute of Pancreatic Diseases, Semmelweis University, Budapest, 1083 Hungary

**Keywords:** Cardiac resynchronization therapy, Sudden cardiac death, Cardiovascular mortality, Scar burden, Cardiac MRI, LGE-CMR

## Abstract

**Aims:**

Late gadolinium enhancement (LGE) assessed by cardiovascular magnetic resonance (CMR) can evaluate myocardial scar associated with a higher risk of sudden cardiac death (SCD), which can guide the selection between cardiac resynchronization therapy with or without a defibrillator (CRT-P/CRT-D). Our aim was to investigate the association between LGE and SCD risk in patients with CRT using the LGE-CMR technique.

**Methods and results:**

We performed a systematic literature search using four databases. The target population was CRT candidates. The primary endpoint was SCD. The risk of bias was assessed using the QUIPS tool.

Fifteen eligible articles were included with a total of 2494 patients, of whom 27%, 56%, and 19% had an implantable cardioverter defibrillator (ICD), CRT-D, and CRT-P, respectively. Altogether, 54.71% of the cohort was LGE positive, who had a 72% higher risk for SCD (HR 1.72; 95% CI 1.18–2.50) compared to LGE negatives. In non-ischemic patients, the proportion of LGE positivity was 46.6%, with a significantly higher risk for SCD as compared to LGE negatives (HR 2.42; 95% CI 1.99–2.94). The subgroup of CRT-only patients showed no difference between the LGE-positive vs. negative candidates (HR 1.17; 95% CI 0.82–1.68). Comparable SCD risk was observed between articles with short- (OR 7.47; 95% CI 0.54–103.12) vs. long-term (OR 6.15; 95% CI 0.96–39.45) follow-up time.

**Conclusion:**

LGE-CMR positivity was associated with an increased SCD risk; however, in CRT candidates, the difference in risk reduction between LGE positive vs. negative patients was statistically not significant, suggesting a role of reverse remodeling. LGE-CMR before device implantation could be crucial in identifying high-risk patients even in non-ischemic etiology.

**Graphical abstract:**

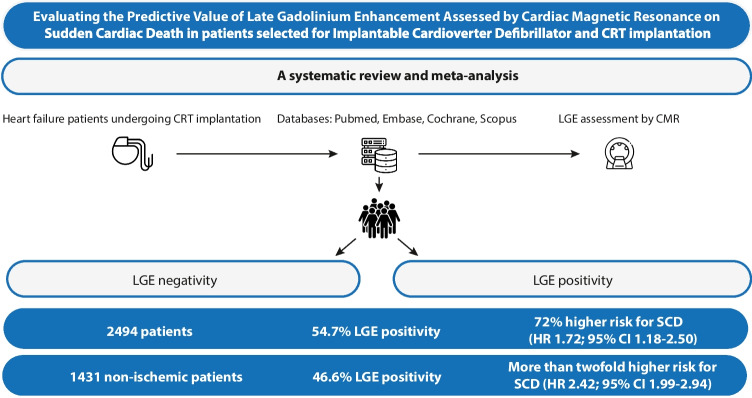

**Supplementary Information:**

The online version contains supplementary material available at 10.1007/s00392-024-02441-2.

## Introduction

Cardiac resynchronization therapy (CRT) is a well-established, effective device therapy for a subgroup of patients who are suffering from heart failure with reduced ejection fraction (HFrEF) and have persistent symptoms of heart failure and wide QRS despite adequate pharmacological therapy [1].

The 5-year mortality rate of patients with HFrEF can reach 50% of which >10% die suddenly [2, 3]. Patients who have suffered heart failure have a six to nine times higher risk of sudden cardiac death (SCD) than the general population [4]. Device therapy such as implantable cardioverter defibrillator (ICD) is an effective SCD treatment, but cardiac resynchronization therapy (CRT) *per se* can also lower the risk of SCD without the defibrillator function by causing reverse remodeling [5].

Late gadolinium enhancement (LGE) is one of the strongest predictors of a subsequent major arrhythmic event such as ventricular tachycardia or ventricular fibrillation (VT/VF) [6]. Modern imaging techniques like cardiac magnetic resonance (CMR) are considered to be the most accurate diagnostic tool for assessing the LGE [7]. In those HFrEF patients eligible for a CRT implantation, the current guidelines [8] recommend an individual risk assessment with CMR to assess the fibrosis when choosing the optimal device between CRT-pacemaker (CRT-P) or CRT-defibrillator (CRT-D). However, there are no specific details given regarding which patients should undergo the examination or to what extent the locations of LGE are deemed high-risk for SCD and require defibrillator implantation.

According to the ESC 2023 Cardiomyopathy Guideline, based on the Task Force’s opinion, the existing level of evidence can support using LGE to guide ICD implantation in subgroups of patients with DCM. Late gadolinium enhancement is observed in 25–35% of patients with DCM, and its presence is a strong risk marker for all-cause mortality and ventricular arrhythmias, both in retrospective and prospective studies [9].

Despite these recommendations, LGE-CMR is not commonly used in routine investigations before CRT implantation in clinical practice, as there may be a considerable financial burden. Therefore, the optimal device selection is based on individual decisions and risk stratification by assessing multiple parameters (primarily age, ischemic etiology, and life expectancy). However, the importance of investigating the LGE-CMR lies in different long-term outcomes of patients and safety events by the device type [8].

Therefore, our aim was to perform a systematic review and meta-analysis of those articles, which investigated the LGE in patients before device implantation (primarily CRT candidates) to assess the relevance and predictive value of LGE for SCD and major arrhythmias, as well as the time dependence of the imposed risk. Additionally, we aimed to define a cut-off value for detecting clinically relevant LGE and to select the high-risk patients by ischemic etiology.

## Methods

This systematic review and meta-analysis was conducted following the recommendations of the Preferred Reporting Items for Systemic Review and Meta-Analyses (PRISMA 2020) guidelines [10] (Supplementary Material Table 1). We registered our research on PROSPERO on 25/11/2022 (ID: CRD42022375597).

### Search strategy

**Fig. 1:**
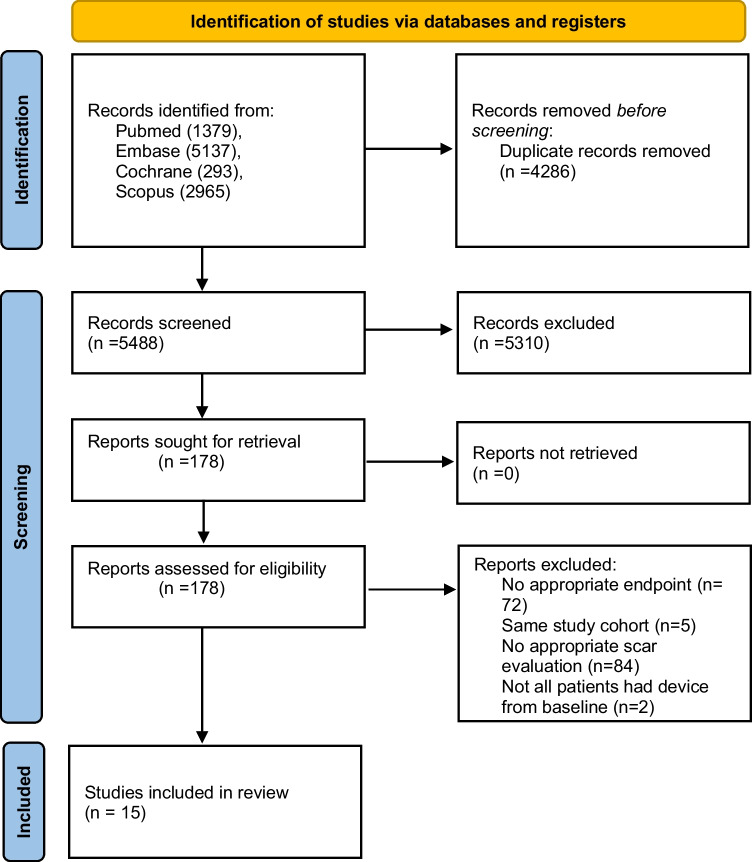
Selection process represented by the PRISMA 2020 flowchart

We used a specific search key with MeSH terms, allowing the search engines thoroughly and systematically focusing on CRT and LGE-CMR, available in the Supplementary Material Section 1. A systematic search was conducted in PubMed, Embase, Scopus, and CENTRAL databases on 18/11/2022 (Fig. 1).

### Inclusion and exclusion criteria

All studies that fit our framework were eligible for the next stage of selection. Randomized controlled trials, non-randomized studies, case reports, case series, prospective or retrospective cohort studies, observational studies, and meta-analysis were found eligible. Following our PFO framework, our patient population was patients with CRT-D or CRT-P. The prognostic factor in question was the presence of LGE assessed by CMR. No restrictions or filters were used in the systematic search. For the full-text selection, we excluded case reports, case series, and meta-analysis.

We also included articles that investigated patients with CRT and ICD because of the limited patient-level data for CRT patients only.

We excluded studies where CRT and CMR were not mentioned, or CMR was mentioned but not used for scar assessment and for safety issues were also excluded. Animal studies, protocols, and studies with no original research data reported, such as reviews, commentaries, letters, and editorials, were excluded.

### Data extraction and quality assessment

All records were imported into a citation management software (EndNote X20). Duplicate removal was performed both automatically and manually by the first author (R.M.). Once duplicate removal had been completed, we started the selection with 3 independent reviewers (R.M., E.ZS., R.E.) in Rayyan Systematic Review Screening Software [11]. Selection was performed according to a pre-existing selection protocol (Supplementary Material Section 2 – Selection protocol) in two phases: title-and-abstract selection and full-text selection. After each phase, Cohen’s kappa was calculated to assess inter-reviewer agreement. An agreement rate of 0.8 was taken as the minimum requirement to move on to the next phase, with disagreements being resolved by consensus or by another reviewer (C.T.). Data collected for extraction included the characteristics of the study, name of the first author, publication year, number of patients, location, participants’ demographics, sample size per group, and follow-up months. Our outcome was sudden cardiac death events with no secondary outcomes.

The Quality in Prognostic Studies (QUIPS) tool was used by two reviewers (R.M. J.B.) who independently assessed the risk of bias based on the recommendation of the Cochrane Collaboration. After the risk of bias assessment, two reviewers (R.M. J.B.) independently assessed the level of evidence certainty GRADE Assessment using GRADE Pro software [12] (Supplementary Material Table 2). Any discrepancy was settled by a third reviewer (C.T.). Different models were used for meta-analysis based on the available reporting of survival analysis. For SCD, most of the results were expressed in terms of HRs and 95% CIs derived from univariate Cox proportional hazards models. We also pooled results expressed via multivariate analysis models. In this way, we analyzed whether any one or more variables had an effect, and if so, to what extent did it affected the observed associations. Multivariate models were adjusted for ejection fraction, age, diabetes, dyslipidemia, smoking, ischemic heart disease, LGE parameters, device type, past syncope, past sustained VT, and the presence of LBBB. When available, competing risk models were used for survival analysis. Results from this approach are presented separately. Fine and Gray proportional subdistribution hazard models and the cumulative incidence function were used in competing risk analysis in the reported few cases [13]. We assessed the odds ratio (OR) based on the reported SCD events separately in the LGE positive and negative groups by Kaplan-Meier graphs using the Shiny-app data-extracting program [14]; we extracted data from the article of Piers et al. [15]. We dichotomized the articles in which SCD event numbers were available, into two groups based on the median value of follow-up times, with short-term follow-up times defined as less than 42 months, and long-term follow-up times defined as longer than 42 months.

### Data analysis

We performed a meta-analysis of studies reporting the same outcome and with comparable ratios. Results were visualized by forest plots. Statistical heterogeneity was analyzed using the *I*^2^ statistic and the Chi^2^ test to acquire probability values; *p*<0.1 was defined to indicate significant heterogeneity. Outcome data and variables for these models were extracted from the eligible articles in accordance with the data extraction plan and were pooled separately within their respective categories. Analysis, visualization, and interpretation of such pooled data did not differ from the methodology described previously. A qualitative and quantitative data synthesis was performed in the case of sufficiently homogenous studies. Data were pooled using the random effects model with the DerSimonien–Laird estimation. In the case of using OR and HR as a measure of effect, *p*-value and 95% confidence interval (CI) were calculated. In order to investigate the presence of LGE, patients were dichotomized as LGE positive or LGE negative patients regardless of the LGE quantification method. Based on LGE assessment, we made 3 different models (univariate, multivariate, and competing risk) with reported hazard ratios (HRs) for SCD events.

## Results

### Article selection and patient characteristics

The search produced 5488 duplicate-free results. A total of 178 articles were sought for retrieval for full-text selection. Altogether, 15 articles were included in this study. Cohen’s kappa was calculated to be 0.85 after the title-and-abstract selection and 1.00 after the full-text selection (Fig. 1).

This study included 11 prospective cohort studies [15–25] and 4 retrospective cohort studies [27–30] published between 2012 and 2022. Altogether, 2494 patients’ data were extracted from the included articles, of whom 27%, 56%, and 19% had ICD, CRT-D, and CRT-P, implanted after CMR, respectively. Altogether, 54.71% of the total population was LGE positive. The mean age of the patients was 62.4±11.5 years, 75.4% were males, and the mean QRS width was 140±30 ms, with a mean ejection fraction of 27.6 ±10.3%. Regarding the comorbidities, slightly more than 50% of patients had hypertension, approximately 42.6% had ischemic etiology, and 24.6% of the total population had diabetes mellitus (Table 1).

**Table 1:** Characteristics of enrolled trials

Author, year	No. of patients	Follow-up time month median (*mean)	Age mean ± SD	Sex female %	QRS width mean ±SD	EF % mean± SD	Ischemic etiology %	ICD %	CRT-D %	CRT-P %	Late gadolinium ehancement (LGE-CMR positive %)	Diabetes %	Hypertension %
Armenta 2012 [16]	78	25*	64±11	-	159±33	22±7	41	0	100	0	54	-	-
Gao 2012 [17]	124	20.7*	61±11	19	136±30	-	48	64	36	0	20.7	24	52
Alexandre 2013 [26]	66	41.5	63±11	3	114±31	23.4±8	100	75	25	0	-	24	42
Fabregat-Andre´s 2013 [18]	103	60	66±12	31	147.3±19	25.8±6	47	0	100	0	50.4	43	56
Piers 2015 [15]	87	45	56±13	38	132±32	29±12	0	48	52	0	63	7	-
Sofia-Alegria 2016 [27]	59	27.1	61±10	-	-	28±6	66	71	29	0	-	-	-
Chaudhry 2017 [28]	60	34.6*	65±9	33	-	27.6±12	43	75	25	0	62	17	58
Acosta 2018 [19]	217	35.5	65±11	18.1	161.5±30	26±8	40	0	71	29	57.6	29.5	-
Barison 2019 [20]	342	20.8	55±15	30	-		0	46	49	5	42	-	-
Bilchick 2019 [21]	100	63.6	65±11	29	157±27	37±16	59	0	100	0	52	33	61
Elming 2019 [22]	108	64.8	60±10	26	131±41	35.7±13	0	57	43	0	48	16.9	-
Travieso Gonzalez 2019 [29]	66	32*	64	37.9	-	25.7	-	50	36	14	-	-	-
Berdibekov 2021 [23]	56	18	-		-	-	-	0	100	0	-	-	-
Sanchez-Somonte 2021 [24]	200	55.2	61±11	18.5	-	20.9±10	52	49	51	0	83	34.5	71
Leyva 2022 [25]	700	83	68±12	17.4	143±27	28±12	58	16	37	47	69	26.4	36.7

### Outcome data

**Fig. 2:**
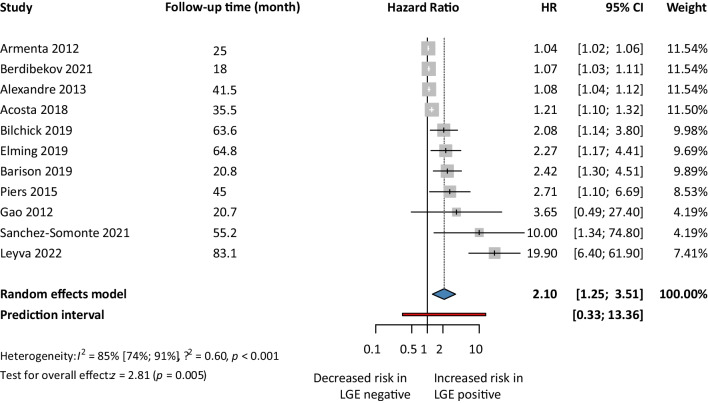
Sudden cardiac death events based LGE-univariate risk model

**Fig. 3:**
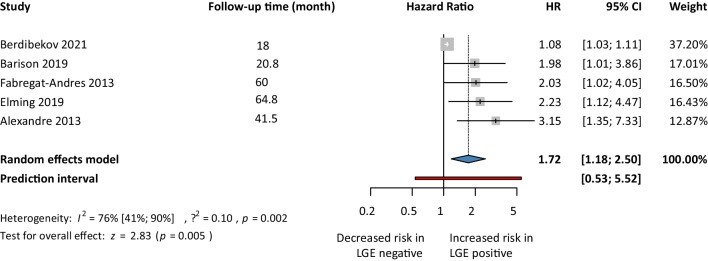
Sudden cardiac death events based on LGE-multivariate risk model

**Fig. 4:**
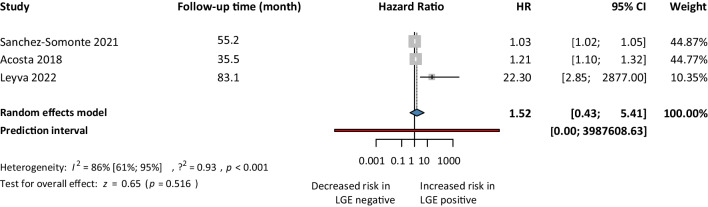
Sudden cardiac death events based on LGE-competing risk model

First, for the univariate risk model, we analyzed 11 articles. Our analysis showed a 2.10. times higher risk of getting SCD events in the LGE positive group compared to the LGE negative group (HR 2.10, 95% CI 1.25–3.51; *p*<0.05) (Fig. 2). Second, we analyzed five articles’ reported HRs for the multivariate risk model. Our analysis showed that the LGE positive group had a 1.72 times higher risk of SCD events compared to the LGE negative group (HR 1.72, 95% CI 1.18–2.50; *p*<0.05) (Fig. 3). Third, we analyzed 3 articles using the competing risk models. Our analysis showed that the risk of developing SCD events in the LGE positive population was almost 52% higher (HR 1.52, 95% CI 0.43–5.41) compared to LGE negative patients, but it was not found to be statistically significant (Fig. 4).

**Fig. 5:**
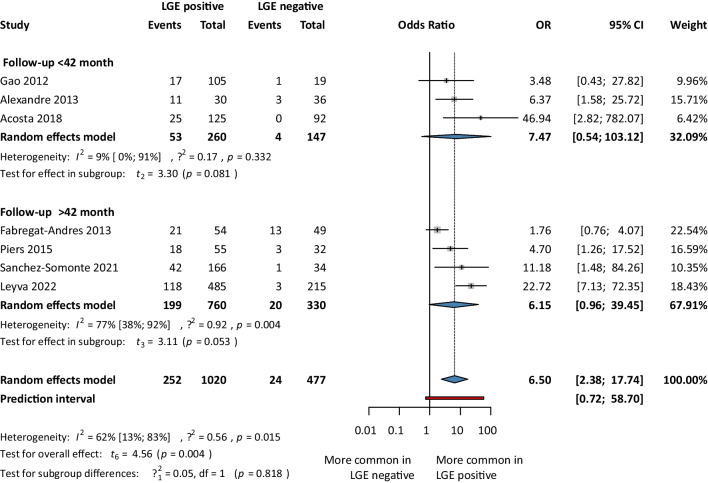
Sudden cardiac death events based on LGE-short and long-term follow-up model; OR, odds ratio

In another analysis, we used odds ratio as the measure of effect where we found 6.5 times higher odds of having SCD events in the LGE positive group compared to the LGE negative group (OR 6.5; 95% CI 2.38–17.74;* p*<0.05). When articles were dichotomized at 42 months, to compare the risk of SCD in those with a short- or long-term follow-up time, there were no differences (OR 7.47; 95% CI 0.54–103.12 and OR 6.15; 95% CI 0.96–39.45, respectively) (Fig. 5).

**Fig. 6:**
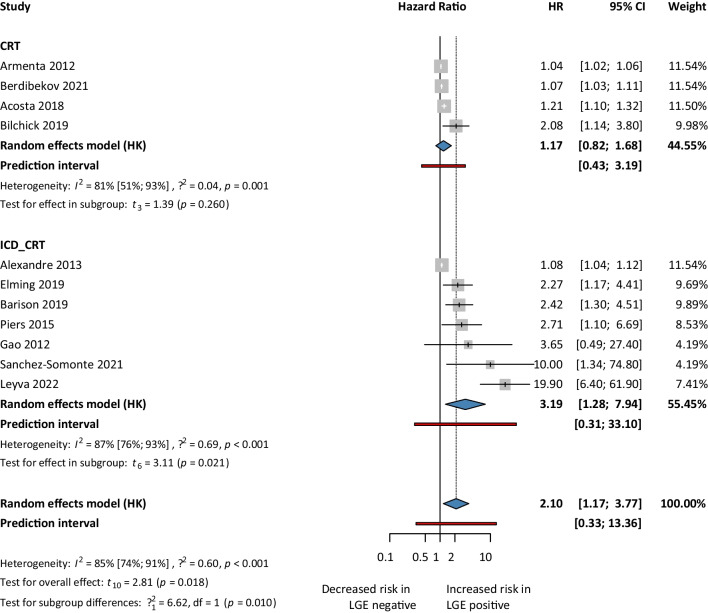
Sudden cardiac death events based on LGE-univariate risk model for CRT vs CRT+ICD

We decided to analyze the subgroup of CRT-only patients since the articles contained CRT-D, CRT-P, and ICD patients as well. In the CRT-only group, there was no statistically significant and clinically relevant difference in the LGE positive and LGE negative patients (HR 1.17; 95% CI 0.82–1.68), while in the mixed population, there was a higher risk for developing SCD events in the LGE positive group (HR 3.19; 95% CI 1.28–7.94; *p*<0.05) (Fig. 6).

Since only one paper reported a mixed population (i.e., CRT-D and CRT-P, Acosta 2018) in the CRT-only group, modifying the weights seemed unreasonable for this analysis. However, we performed a sensitivity analysis by excluding the above-mentioned paper and obtained numerically similar results (HR 1.21; 95% CI 0.53–2.75) (Supplementary material – Supplementary Figure 2).

**Fig. 7:**
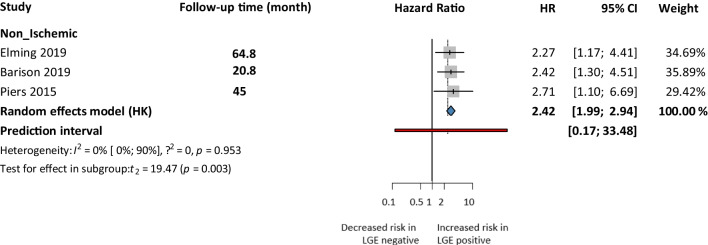
Sudden cardiac death events based on LGE-univariate risk model for non-ischemic subgroup

We performed a subgroup analysis for non-ischemic cardiomyopathy (NICM) patients with 3 articles, where we found a significant, 2.42 times higher risk of SCD events in the LGE positive group compared to the LGE negative (HR 2.42 CI: 1.99–2.94). The LGE positivity ratio was 51% in this subgroup compared to 54.71% of the total cohort (Fig. 7). Additionally, we attempted to describe the characteristics of non-ischemic patients with LGE. Based on a comparison, (22) rather male patients and those with a long-term history of cardiomyopathy showed LGE positivity.

For risk of bias assessment, we used the QUIPS tool as the standard prognostic study tool. Based on the six different domains, a high risk of bias was detected in four articles (Barison [20], Berdibekov [23], Sofia-Alegria [27], Travesio [29]) (Supplementary Figure 1). All these articles were reported as only conference abstracts. We found two articles with a low risk of bias (Acosta [19] and Leyva [25]). All the other ten articles were considered as having a moderate risk of bias.

## Discussion

This current meta-analysis shows that the presence of LGE in HFrEF patients eligible for ICD or CRT correlated with the incidence of future SCD events. LGE positive patients had a 2–6 times higher risk of developing malignant arrhythmias than those without LGE, in whom the number of SCD events was negligible. In a subgroup of patients undergoing CRT implantation, the difference in relative risk reduction between LGE positive vs. negative patients was statistically not significant, suggesting a significant role of CRT-induced reverse remodeling in decreasing the overall risk of SCD. Additionally, in the subset of non-ischemic patients, the occurrence of LGE positivity was not negligible and their SCD risk was comparable to the overall population.

In general, the presence of LGE is a relevant parameter associated with the incidence of malignant arrhythmias and SCD events [30] as it was previously described in several prospective studies and a recent meta-analysis. However, specific data is scarce on HFrEF patients with wide QRS who are candidates for a CRT device. At the same time, the current guidelines recommend to assess LGE-CMR before their device selection, but no further suggestions are stated for selecting those patients in whom the evaluation of LGE would significantly influence the choice between CRT-P vs. CRT-D implantation and thereby the patients’ outcome and safety [8].

In the current analysis, our initial aim was to investigate the overall SCD risk in CRT candidates by the presence of LGE, which showed that LGE positivity was associated with a substantially higher risk on SCD. At the same time, CRT patients may have a lower subsequent risk of SCD compared to those referred to an ICD, as CRT *per se* can decrease the risk of malignant arrhythmias by inducing reverse remodeling. However, the development of subsequent reverse remodeling is multifactorial (e.g., QRS morphology, location of the LV lead) [31–33], in which the presence and amount of scar is also determinative [34]. Previous studies on medical treatments and device therapies also revealed that those which induce left ventricular reverse remodeling are associated with a lower risk of SCD events [3]. This finding is supported by the BUDAPEST CRT Upgrade trial where the incidence of VT/VF events was significantly lower in those patients implanted with CRT-D devices, which signals the possible role of the effect of reverse remodeling [35, 36]. Additionally, SGLT-2 inhibitors and sacubitril/valsartan were also associated with a reduced risk of SCD and arrhythmia burden primarily by their effect on cardiac remodeling [37]. Vericiguat, a stimulator of soluble guanylate cyclase, also proves to be a promising candidate of medications reducing SCD risk [38].

Additionally, we aid to identify those high-risk CRT candidates, who can have a benefit by adding an ICD to CRT. First, when the incidence and proportion of LGE positivity in HFrEF patients before device implantation were investigated, we found that data varied widely in the literature, in patients with ischemic etiology between 90 and 100% and in non-ischemic patients 40 and 70%, respectively, showing 48% in the current cohort. However, the wide range is associated with several factors such as the characteristics of the investigated populations; these results are clearly reflecting that the risk of having LGE is higher in patients with ischemic etiology. Therefore, in everyday clinical practice, they are preferred to be implanted with a CRT-D device [39–41]. However, patients with non-ischemic etiology are still a matter of debate. Based on previous milestone trials (e.g., DANISH), it is proved that certain subset of non-ischemic patients may have a mortality benefit from an ICD backup (e.g., younger ones) [42]. As we described in the current meta-analysis, the occurrence of LGE positivity in non-ischemic patients was approximately 46%, and their SCD risk was comparable with overall HFrEF candidates for ICD/CRT; therefore, identifying the high-risk patients with a NICM is crucial. However, data was limited, and it seems rather male patients who have a long-term history of cardiomyopathy have a higher risk to develop LGE with non-ischemic etiology.

**Fig. 8:**
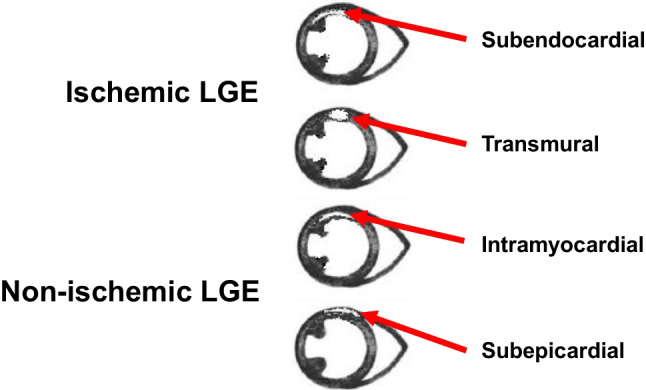
Typical examples of ischemic and non-ischemic LGE

Our initial aim was also to detect an optimal cut-off value of LGE, above which a significantly higher SCD risk could be observed, but only two articles reported such data. Acosta et al. [19] found that scar mass >10 g had 100% sensitivity, 72% specificity, and 30.1% positive predictive value, while scar mass <10 g had 100% negative predictive value for the occurrence of appropriate ICD therapy. At the same time, Leyva et al. [25] identified the best predictor of arrhythmic endpoints as a threshold of >17 g with border zones. Meanwhile, in a less heterogeneous population of patients with hypertrophic cardiomyopathy, Greulich et al. [43] found an LGE amount of >5% left ventricular (LV) mass anticipates the highest risk for SCD, which may indicate an ICD implantation. These data also suggest that technical questions should be clarified beyond the localization for both ischemic and non-ischemic LGE (Fig. 8). Upon positive LGE detection by CMR, further assessment can be made using precision techniques, such as T1 mapping [44]. Kolentinis et al. compared LGE vs. native and post-contrast T1 mapping, and they found that LGE remains the method of choice for ischemic scar quantification as native T1 mapping underestimated scar area while post-contrast T1 overestimated it [45].

Based on these results, further prospective trials are warranted such as PROFID-EHRA in order to properly select those CRT candidates who may benefit from performing a CMR before CRT implantation, therefore to predict their SCD risk and also aid the optimal device selection [46]. The RESET-CRT project is a retrospective observational study demonstrating that in patients who have indication for CRT, the implantation of a CRT-P is not inferior to CRT-D regarding all-cause mortality, as a prelude to the ongoing randomized controlled RESET-CRT study [47].

Another promising ongoing RCT is the BRITISH trial which will assess whether the use of scar detection based on CMR to direct devices implantation with NICM and an LVEF ≤35% is associated with a reduction in mortality [48].

The CMR-ICD aims to compare OMT vs. ICD in NICM with HFrEF, of which results are eagerly awaited, just like the CRT-REALITY study, in which NICM and LVEF ≤35% patients are randomized to CRT-P or CRT-D implantation based on LGE on CMR [49].

These ongoing trials clearly show the high relevance of the topic and hopefully will contribute to the optimal patient selection for CMR before CRT implantation.

## Limitations

Our meta-analysis is the first one to focus on CRT patients and on SCD prediction by LGE assessment by CMR. Recent meta-analyses were focused on ICDs only or showed *mixed* populations [50]. However, investigating those patients selected for CRT implantation revealed that CRT *per se* decreases the risk of SCD; therefore, the presence of LGE and predicting subsequent reverse remodeling are relevant in selecting the optimal device.

Our meta-analysis has some limitations. First, a limited number of publications enrolled only CRT candidates. Second, sudden cardiac death events were not exactly defined in all papers. Third, some articles reported only univariate, others multivariate, and competing risk models, which limited the number of papers within the different subgroups.

Out of the 15 articles, 4 articles were only conference abstracts with limited information and poor risk of bias evidence. Only observational studies were included in our analysis; no RCTs were available.

Special cardiomyopathies like HCM and restrictive phenotypes caused by amyloidosis or sarcoidosis were not declared to exclude in all articles.

Another limitation is that CRT patients may have differed in terms of age, disease, and SCD risk factors as the included articles were mainly observational studies, and not all reported a baseline clinical characteristic table for the CRT and ICD subgroups, so CRT patients may had lower risk at baseline compared to ICD patients.

We wanted to identify a cut-off value for the LGE as well as responders vs.non-responders and a CRT-P vs. CRT-D subgroup analysis which was not feasible due to limited data. Only the subgroups of non-ischemic patients could be investigated, where the differences of the baseline clinical characteristics by the presence of LGE were described in the manuscript of Elming et al. [22].

Results of the mixed populations may have been affected by the ICD function of CRT-D patients in the CRT-only group as there were 400 CRT-P and 1108 CRT-D patients in the CRT cohort (Fig. 6).

## Conclusions

In HFrEF patients selected for device implantation, the presence of LGE correlated with the incidence of subsequent SCD events. LGE positive patients had a substantially higher risk of developing malignant arrhythmias compared to those without LGE, in whom the number of SCD events was negligible. In patients undergoing CRT implantation, the difference in relative risk reduction between LGE positive or negative patients was statistically not significant, suggesting a relevant role of CRT-induced reverse remodeling in decreasing the overall risk of SCDs. Our results suggest that CMR prior to device implantation could be important for certain high-risk subgroups even with non-ischemic etiology.

## Supplementary Information

Below is the link to the electronic supplementary material.

Supplementary file1 (DOCX 336 KB)

## Data Availability

All data used in this meta-analysis is publicly available. Please contact the corresponding author for any and all requests regarding the datasets.
