# Supplementary material for: Evaluating the predictive value of late gadolinium enhancement assessed by cardiac magnetic resonance on sudden cardiac death in patients selected for implantable cardioverter defibrillator and cardiac resynchronization therapy implantation: a systematic review and meta-analysis
Source: Clin Res Cardiol. 2024 Apr 8;115(4):523–35. doi: 10.1007/s00392-024-02441-2 (PMC13013130; doi:10.1007/s00392-024-02441-2)
Supplement: Supplementary file 1 — Supplementary file1 (DOCX 336 KB) [file 392_2024_2441_MOESM1_ESM.docx]

**Supplementary information**

**Supplementary Figure 1-** QUIPS tool used for risk of bias assessment for endpoint


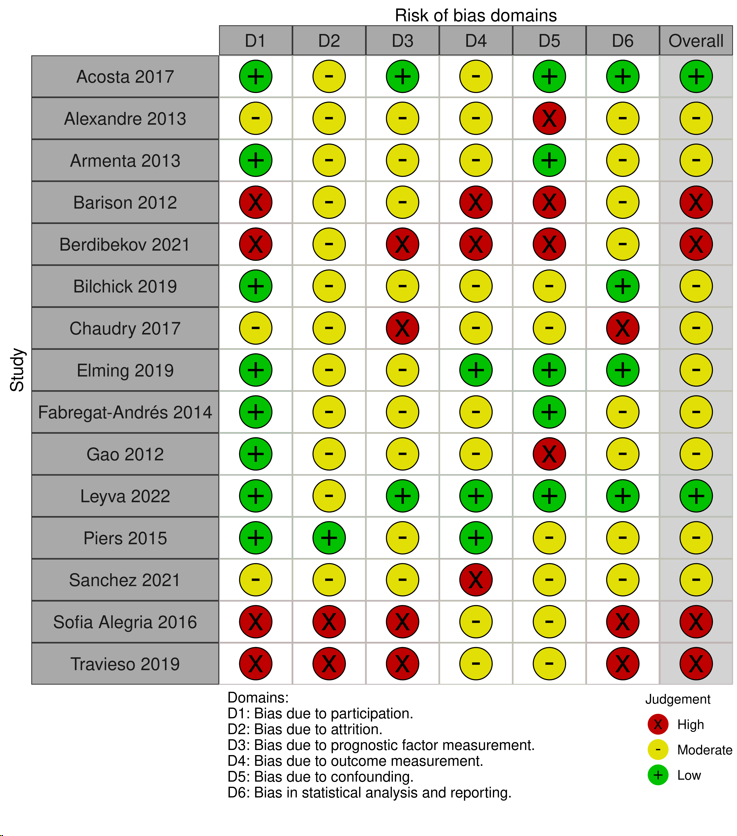


**Supplementary Figure 2**- Sudden cardiac death events based on LGE- univariate risk model for CRT-D vs CRT+ICD


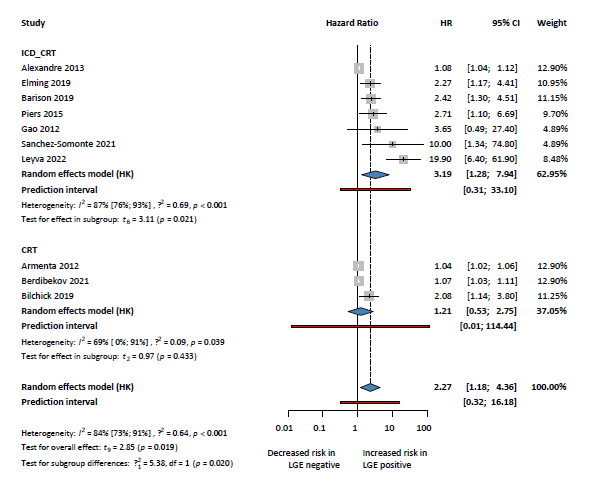


Supplementary Table 1 - PRISMA checklist

| **Section and Topic** | **Item #** | **Checklist item** | **Location where item is reported** |
| --- | --- | --- | --- |
| **TITLE** | | |  |
| Title | 1 | Identify the report as a systematic review. | Title |
| **ABSTRACT** | | |  |
| Abstract | 2 | See the PRISMA 2020 for Abstracts checklist. | Abstract |
| **INTRODUCTION** | | |  |
| Rationale | 3 | Describe the rationale for the review in the context of existing knowledge. | Section 1 |
| Objectives | 4 | Provide an explicit statement of the objective(s) or question(s) the review addresses. | Section 1 |
| **METHODS** | | |  |
| Eligibility criteria | 5 | Specify the inclusion and exclusion criteria for the review and how studies were grouped for the syntheses. | Section 2.2  Supplementary material selection protocol |
| Information sources | 6 | Specify all databases, registers, websites, organisations, reference lists and other sources searched or consulted to identify studies. Specify the date when each source was last searched or consulted. | Section 2.2 |
| Search strategy | 7 | Present the full search strategies for all databases, registers and websites, including any filters and limits used. | Section 2.1 |
| Selection process | 8 | Specify the methods used to decide whether a study met the inclusion criteria of the review, including how many reviewers screened each record and each report retrieved, whether they worked independently, and if applicable, details of automation tools used in the process. | Section 2.3  Supplementary material selection protocol |
| Data collection process | 9 | Specify the methods used to collect data from reports, including how many reviewers collected data from each report, whether they worked independently, any processes for obtaining or confirming data from study investigators, and if applicable, details of automation tools used in the process. | Section 2.3 |
| Data items | 10a | List and define all outcomes for which data were sought. Specify whether all results that were compatible with each outcome domain in each study were sought (e.g. for all measures, time points, analysis), and if not, the methods used to decide which results to collect. | Section 2.3 |
|  | 10b | List and define all other variables for which data were sought (e.g. participant and intervention characteristics, funding sources). Describe any assumptions made about any missing or unclear information. | Section 2.4 |
| Study risk of bias assessment | 11 | Specify the methods used to assess risk of bias in the included studies, including details of the tool(s) used, how many reviewers assessed each study and whether they worked independently, and if applicable, details of automation tools used in the process. | Section 2.3  ,2.4 |
| Effect measures | 12 | Specify for each outcome the effect measure(s) (e.g. risk ratio, mean difference) used in the synthesis or presentation of results. | Section 2.4 |
| Synthesis methods | 13a | Describe the processes used to decide which studies were eligible for each synthesis (e.g. tabulating the study intervention characteristics and comparing against the planned groups for each synthesis (item #5)). | Section 2.4 |
|  | 13b | Describe any methods required to prepare the data for presentation or synthesis, such as handling of missing summary statistics, or data conversions. | Section 2.4 |
|  | 13c | Describe any methods used to tabulate or visually display results of individual studies and syntheses. | Section 2.4 |
|  | 13d | Describe any methods used to synthesize results and provide a rationale for the choice(s). If meta-analysis was performed, describe the model(s), method(s) to identify the presence and extent of statistical heterogeneity, and software package(s) used. | Section 2.4 |
|  | 13e | Describe any methods used to explore possible causes of heterogeneity among study results (e.g. subgroup analysis, meta-regression). | Section 2.4 |
|  | 13f | Describe any sensitivity analysis conducted to assess robustness of the synthesized results. | Section 2.4 |
| Reporting bias assessment | 14 | Describe any methods used to assess risk of bias due to missing results in a synthesis (arising from reporting biases). | Section 2.4 |
| Certainty assessment | 15 | Describe any methods used to assess certainty (or confidence) in the body of evidence for an outcome. | Section 2.3 |
| **RESULTS** | | |  |
| Study selection | 16a | Describe the results of the search and selection process, from the number of records identified in the search to the number of studies included in the review, ideally using a flow diagram. | Section 3.2  Figure 1 |
|  | 16b | Cite studies that might appear to meet the inclusion criteria, but which were excluded, and explain why they were excluded. | Figure 1 |
| Study characteristics | 17 | Cite each included study and present its characteristics. | Section 3.1  Table 1 |
| Risk of bias in studies | 18 | Present assessments of risk of bias for each included study. | Section 3.2  Supplementary Figure 1 |
| Results of individual studies | 19 | For all outcomes, present, for each study: (a) summary statistics for each group (where appropriate) and (b) an effect estimate and its precision (e.g. confidence/credible interval), ideally using structured tables or plots. | Section 3.2  Figure 5  Supplementary Figure 1 |
| Results of syntheses | 20a | For each synthesis, briefly summarise the characteristics and risk of bias among contributing studies. | Section 3.2  Supplementary Figure 1 |
|  | 20b | Present results of all statistical syntheses conducted. If meta-analysis was done, present for each the summary estimate and its precision (e.g. confidence/credible interval) and measures of statistical heterogeneity. If comparing groups, describe the direction of the effect. | Section 3.2  Figure 5 Supplementary Figure 1 |
|  | 20c | Present results of all investigations of possible causes of heterogeneity among study results. | NA |
|  | 20d | Present results of all sensitivity analysis conducted to assess the robustness of the synthesized results. | NA |
| Reporting biases | 21 | Present assessments of risk of bias due to missing results (arising from reporting biases) for each synthesis assessed. | Section 3.2  Supplementary Figure 1 |
| Certainty of evidence | 22 | Present assessments of certainty (or confidence) in the body of evidence for each outcome assessed. | Table 1. |
| **DISCUSSION** | | |  |
| Discussion | 23a | Provide a general interpretation of the results in the context of other evidence. | Section 4.1 |
|  | 23b | Discuss any limitations of the evidence included in the review. | Section 4.3. |
|  | 23c | Discuss any limitations of the review processes used. | Section 4.3 |
|  | 23d | Discuss implications of the results for practice, policy, and future research. | Section 4.2 |
| **OTHER INFORMATION** | | |  |
| Registration and protocol | 24a | Provide registration information for the review, including register name and registration number, or state that the review was not registered. | Section 2 |
|  | 24b | Indicate where the review protocol can be accessed, or state that a protocol was not prepared. | Section 2 |
|  | 24c | Describe and explain any amendments to information provided at registration or in the protocol. | Section 2.1 |
| Support | 25 | Describe sources of financial or non-financial support for the review, and the role of the funders or sponsors in the review. | Funding |
| Competing interests | 26 | Declare any competing interests of review authors. | Conflict of interest |
| Availability of data, code and other materials | 27 | Report which of the following are publicly available and where they can be found: template data collection forms; data extracted from included studies; data used for all analysis; analytic code; any other materials used in the review. | Data availability |

Supplementary Material Table 2- GRADE of the systematic review and meta-analysis.

| **№ of studies** | **Certainty assessment** | | | | | | **Effect** | | | **Certainty** | **Importance** |
| --- | --- | --- | --- | --- | --- | --- | --- | --- | --- | --- | --- |
|  | **Study design** | **Risk of bias** | **Inconsistency** | **Indirectness** | **Imprecision** | **Other considerations** | **№ of events** | **№ of individuals** | **Rate (95% CI)** |  |  |
| sudden cardiac death (assessed with: Sudden cardiac death events) | | | | | | | | | | | |
| 16 | observational studies | serious | serious^a^ | serious | not serious | strong association | 487 | 2624 | event rate 95.0% (2.38 to 17.74) | ⨁⨁◯◯ Low | CRITICAL |

Supplementary Material Section 1. –Search key

((Myocardial Fibrosis) OR (Scar Characterization) OR (scar burden) OR (late gadolinium enhancement) OR (LGE) OR (Magnetic resonance imaging) OR (MRI) OR (cardiovascular magnetic resonance)) AND ((Cardiac Resynchronization Therapy) OR (CRT))

Supplementary Material Section 2. –Selection protocol:

Predictive value of scar burden assessed by cardiac MRI on sudden cardiac death in cardiac resynchronization therapy patients

Clinical question: What is the association between the degree of scar burden assessed by cMR and sudden cardiac death in CRT patients?

Patients: heart failure patients undergoing CRT

Prognostic factor: Scar burden assessed by cardiac MRI

Primary Outcomes: sudden cardiac death

TITLE AND ABSTRACT:

Inclusion:

- Randomized controlled trials, non-randomized clinical trials, case reports, case series, prospective or retrospective cohort studies, observational studies, meta-analysis will be included

- We are looking for patients with cardiac resynchronization therapy (CRT)

It can be CRT-D and CRT-P as well, also called biventricular pacemaker

- We will include studies where Cardiac MRI was used for patients to scar assessment

-It can be scar mass, scar %,core mass, GZF( gray zone fibrosis),

- Papers with no abstracts, no titles or no authors are to be included for full text selection

- For those articles where there are more articles from the same author with the

same research all articles should be included to avoid discrepancy between the the selection

Exclusion:

- Studies where CRT or MRI not mentioned are for exclusion

-MRI mentioned but not used for scar assessment but for safety issues

-Animal studies, protocols, studies with no authentic evidence reported such as reviews,

commentaries, letters, editorials, conference or meeting abstracts will be excluded

FULL TEXT

Inclusion:

- Randomized controlled trials, non-randomized clinical trials, prospective or retrospective cohort studies, observational studies, will be included

- We include abstract only as well.

- We will include studies where MRI used for scar assessment or to evaluate myocardial fibrosis or LGE-CMR, late gadolinium enhancement is mentioned

- Our primary endpoint is sudden cardiac death, so in the full text we have to search for appropriate shock, VT, VF ventricular arrhythmia, or SCD. If there are a number of these events that happened during the follow-up time we include, if not then we don’t.

- for data extraction we need numbers about the outcome, scar evaluation, (LGE-CMR or scar mass, scar %) number of CRT patients

- We can include studies investigating responderity but just in case it includes the following things mentioned above.

Exclusion:

-Animal studies

-Colorectal cancer or any other cancer, tumor can be excluded at once.

-NO scar assessment

-NO arrhythmic endpoint

About multiple publications using the same patient population:

- In the full-text phase you will have to assess whether there are eligible publications/

abstract presenting data from overlapping or completely identical populations

- In this case, only one can be included in your quantitative synthesis

- If they report and additional outcome, it is okay to use them for that outcome

- We mainly choose the one with the highest patient number (or best quality regarding the available data)
